# “They Can’t Possibly Understand What I’m Going Through”: Female Farmers’ Perspectives on Barriers to Care in Georgia

**DOI:** 10.3390/ijerph21091130

**Published:** 2024-08-27

**Authors:** Noah Hopkins, Lauren Ledbetter Griffeth, Chase Reece, Christina Proctor

**Affiliations:** 1 Department of Health Promotion and Behavior, University of Georgia, Athens, GA 30602, USA; chase.reece@uga.edu (C.R.); cproctor@uga.edu (C.P.); 2 Office of Learning and Organizational Development, University of Georgia, Athens, GA 30605, USA; lauren1@uga.edu

**Keywords:** barriers to care, female farmers, stigma, rural healthcare, farm identity

## Abstract

The purpose of this study was to explore female farmers’ perspectives on barriers to engaging with resources for physical and mental healthcare faced by agriculture producers in the state of Georgia. In-depth interviews were conducted with female farm owners and managers (*n* = 16) across the state. Interviews were recorded and transcribed, and researchers coded interviews separately before thematic analysis was used to identify common themes. Three primary themes were identified: (i) formal healthcare challenges, (ii) stigma, and (iii) cultural norms. Formal healthcare challenges included time constraints, healthcare costs, and a lack of cultural competence from healthcare providers. Both community and self-stigma were identified as barriers to engaging with mental health resources. Cultural norms that acted as a barrier to care included the prioritization of farm operations, self-reliance, pride, and the minimization of health concerns. Interviewees identified gender differences in the impact of stigma and cultural norms, reporting that these sociocultural barriers were more prominent among older, male producers. Central to many of these barriers is the concept of ‘farm identity’, where farmers’ commitment to their operations consistently trumped concerns about physical or mental health. Future efforts to improve health outcomes among farmers should utilize the concept of farm identity as a guide for tailoring interventions and improving cultural competence among rural healthcare providers.

## 1. Introduction

Farming is an occupation characterized by a high risk of physical injury due to the regular use of heavy machinery and interactions with livestock, as well as exposure to hazardous chemicals [1]. Consistently ranked as one of the most dangerous occupations in the United States, there were 777 fatal injuries in the agricultural field between 2017 and 2021, and the industry’s non-fatal injury rate is 4.1 per 100 workers, compared to the average rate of 2.7 per 100 workers for all occupations in 2022 [2]. Reporting indicates that between 2015 and 2019, over 60,000 individuals received emergency medical treatment for injuries attributed to agricultural work, although these figures may be severely underestimated due to under-reporting, indicating that occupational health issues may have a higher prevalence than is currently understood [3]. In addition, farmers face a multitude of stressors that may impact their mental health, including weather and environmental concerns, political and commodity market fluctuations, financial cost of operations, and isolation from community members and healthcare resources [4,5,6].

Farmers living in rural areas must contend with fewer healthcare providers per capita, greater distance from healthcare resources, and lower rates of insurance coverage than the general population [7,8,9]. Farmers are less likely to receive health insurance through their employer or from federal programs, which exposes them to a greater risk of financial hardship due to medical expenses [10]. This disparity is more pronounced for mental healthcare resources, as it is estimated that roughly 60% of rural adults live in an area experiencing a deficit in mental health services and providers [11].

Previous research has identified numerous barriers to engaging with healthcare resources in the farming community. Formal healthcare challenges associated with limited insurance coverage, distance from specialized services, and poor perceived quality of care have been documented in farmers both in the United States and internationally [12,13,14]. Even when healthcare resources are available, research among farmers indicates there are several cultural barriers that prevent farmers from readily engaging with them. In prior work, farmers reported that a preference for self-reliance and perceived pressure to maintain the image of the ‘resilient worker’ may prevent engagement with healthcare resources [12,13,14,15]. Cultural barriers to help-seeking behavior are more prominent for mental health challenges, and farmers have identified significant concerns about stigma associated with help-seeking for mental health challenges, specifically that they would be perceived as weaker than their peers, and potentially unfit to continue working in agriculture [6,12,13].

While agriculture in the United States remains a male-dominated occupation, the proportion of female agricultural producers has been steadily increasing, with a recent census identifying over 1.2 million female producers, roughly 36% of all farmers, in 2022 [16]. This shift in producer demographics highlights a need for a deeper understanding of the experience of female agriculture producers, particularly when trying to engage with resources for physical or mental healthcare. While female farmers have been included in prior qualitative studies exploring barriers to engaging with healthcare, few publications have focused specifically on exploring female producers’ perspectives on barriers to engaging with care [6,17,18,19]. Research indicates that women working in male-dominated occupations may adopt conventionally masculine value systems and behaviors to adapt to their work environment, but the extent to which this impacts intent to engage in help-seeking behavior among female farmers is unknown [20,21]. A better understanding of perceived barriers to engaging with healthcare resources among female farmers in the United States is critical for the development of appropriate educational or behavioral interventions targeting this subset of the agricultural community.

## 2. Methods

This study collected data from 16 female farm owners and managers. Respondents resided in 11 geographically dispersed counties across the state of Georgia. The majority of Georgia is rural, with 124 of the state’s 159 counties being classified as rural [22].

### 2.1. Participant Recruitment

Farm women were recruited to participate in 35 min to 1 h interviews, and all but three participants were full-time farmers. The research team (principal investigator and two graduate students) operationally defined full-time farming as deriving 75% or more of their income from farm operations. This definition was chosen to ensure that respondents were primarily earning a living from their farm operations and could speak to the experience of this occupation as opposed to being engaged in small-scale hobby farming. One part-time farmer held a position in a different field, and the other two, one of whom was married to a farmer, held jobs in the agricultural field with one working towards farming full-time. Researchers recruited potential participants by connecting with farmers via Cooperative Extension (Cooperative Extension is an organization that translates and communicates academic research to a broader audience within the State of Georgia, bridging the gap between academic research and community members. Cooperative Extension is the University of Georgia’s primary method of communicating scientific findings to Georgia’s agricultural producers and rural communities), sharing interest forms at farm-related events, and asking prior participants to use word-of-mouth to refer other female producers to the research team. If a prospective interviewee completed the questionnaire on the interest forms, then the research team reached out to them to schedule their appointment. The use of multiple methods to recruit participants contributed to the diversity of commodities and perspectives represented in these findings.

### 2.2. Data Collection

Researchers traveled to 15 farms to conduct semi-structured interviews with farmers, and one interview was conducted online using Zoom (Version 5.17.10). Researchers used a script to maintain consistency of interview procedures, and the same three researchers, two of whom had conducted and published prior qualitative research in the farming community, facilitated all interviews to keep the methodology consistent across appointments. After providing verbal and written informed consent, interview participants were asked a series of 18 questions about their experiences working in agriculture, accessing healthcare resources, and managing stress. Interview questions included “If you have experienced any negative health effects as a consequence of farming, were you able to receive treatment?”, “Is there an existing treatment option for mental health issues or substance abuse in your area?”, and “Would you feel comfortable discussing mental health issues that you are experiencing with your family doctor or current healthcare provider?” Researchers asked follow-up questions to explore responses in more detail. Prior to starting the interview, researchers informed participants that participation in this study was completely voluntary, and that they had the ability to decline to answer a specific question or stop the interview at any time. All participants completed their interview in its entirety, and no participants declined to respond to any questions asked during the interview process. Participants were compensated for their participation with a USD 50 gift card, and no participants withdrew from the study. Saturation was reached over the course of the 16 interviews conducted by the research team. This study was approved by the institutional review board. The full interview script used for data collection has been included as Supplementary Materials to this manuscript.

### 2.3. Data Analysis and Interpretation

Interviews were recorded before being transcribed using Trint, an online service. After the transcripts were generated, one of the graduate students involved in the project cross-checked the generated transcripts against the audio recordings to ensure that participant responses were accurately recorded and that all identifying information was removed. The three researchers used a combination of inductive and deductive coding to analyze the data [23]. While the initial coding dictionary was developed following a preliminary review of the transcripts by the researchers, with a framework to categorize participant responses, the interviewers used participant responses to determine themes and subthemes present in the interview data itself. The same three researchers who facilitated the interview process then coded the data question-by-question, before consolidating responses across questions into broader themes and subthemes (e.g., discussions of time constraints when engaging with healthcare resources were categorized as the ‘time’ subtheme of the broad theme ‘Formal Healthcare Challenges’).

## 3. Results

### 3.1. Participant Demographics

A total of 16 farmers living in 11 counties across the state were interviewed. All respondents were female, and most respondents were White (81.25%). Of the three non-White respondents, two identified as African American, and one identified as Latina. Respondents had maintained their operations for a wide range of years, with experience ranging from three to 25 years in this sample. Similarly, there was a wide range of generations of farmers in this sample, with most respondents indicating that they were second- or third-generation farmers (53.33%). Most farmers ran diversified operations, and numerous commodities were represented in this sample, including beef cattle, dairy, citrus, poultry, ornamental crops, and strawberries. The characteristics of each participant can be seen in Table 1.

### 3.2. Health Outcomes

Almost all participants reported experiencing negative health outcomes that they attributed to working in agriculture. Interview participants reported several different physical injuries attributed to farm work, including repetitive stress injuries, joint pain in the knees and back, muscle tears, broken bones, and experiences with heat exhaustion and heat stroke. Participants also identified several mental health challenges attributed to farming, including high levels of stress resulting in generalized anxiety, anxiety attacks, and sleep disturbance, as well as depression, PTSD (Post Traumatic Stress Disorder), and burnout. Just under half of respondents reported feeling comfortable discussing mental health challenges with their family doctor, with others expressing a preference for a specialist or concerns about confidentiality as barriers.

### 3.3. Barriers to Help-Seeking

Three major themes were identified as barriers to engaging with care in the farming community: (i) formal healthcare challenges, (ii) stigma, and (iii) cultural norms. Interview participants offered the most first-hand experience with formal healthcare challenges, and throughout interviews, detailed how both stigma and cultural norms acted as barriers to help-seeking behavior among farmers that they worked with and knew. Longer relevant quotes can be seen in Table 2.

### 3.4. Formal Healthcare Challenges

#### 3.4.1. Time Constraints

During interviews, farmers reported numerous barriers to accessing healthcare from a structural or healthcare provision standpoint. The scarcity of resources in rural areas resulted in both long travel times and delays in being able to access care, with many farmers indicating that they had to wait for months after an incident to engage with a healthcare provider. When asked about accessibility of care in their area, one farmer located in Southwest Georgia said, “it’s, um, arguably 45 min to a functioning ER (emergency room) that can handle some nontraditional stuff. Is that okay? Probably not”. The same farmer reported having to wait six months for a diagnostic appointment due to high demand and limited providers of specialized services in their area. Another interviewee noted that while there was a healthcare resource in her area, when she injured her shoulder, a provider said that he could not touch her due to his religious beliefs and that she had to travel a further 20 miles to receive treatment.

Time away from farming operations was identified as a significant barrier by several farmers, who reported that taking hours out of the day to either see a service provider or recover from a procedure was massively disruptive. One farmer with a small operation stated that it would be too challenging to find assistance that would make it feasible for her or her partner to take time away from the farm to recover. Another stated that long waiting periods to receive care that conflicted with regular farm activity, such as feeding animals or cleaning stalls, made it incredibly challenging to schedule appointments or seek care. When asked how time impacted their ability to engage with healthcare resources, one farmer said, “if you go into the ER and you tell them (farmers) ‘oh it’s going to be six hours’, that’s the next feeding or milking. They Would be like ‘I’m sorry, I’ve got to go home and feed my cows. I can’t sit in the waiting room and wait six hours’”.

#### 3.4.2. Cost of Accessing Care

Another barrier frequently identified by farmers was the cost of care—interviewees frequently reported the cost of either healthcare services or health insurance itself as a barrier to engaging with services. One farmer said, “It was actually cheaper to pay (for a major surgery) out of pocket than to have insurance. So I got lucky”. Another stated “…a lot of them are scared of how much it’ll cost. A lot of farmers don’t have health insurance. We have health insurance because my mom’s a teacher, but a lot of people don’t. If you have a wife that works on the farm too, health insurance, to buy it yourself, is ridiculously expensive”. Another farmer illustrated how a lack of insurance could be compounded by other farm considerations, such as time and labor demands, by saying, “I don’t have great health insurance right now…or the time, it doesn’t feel like I can really make an appointment, especially when folks are calling out sick…Like we harvest pretty much every day”. Even when farmers tried to secure health insurance to offset the cost of healthcare utilization, they were often stymied by logistical hurdles that took time away from their operations and prevented them from activating coverage.

#### 3.4.3. Provider Disconnection and Cultural Competence

Challenges activating health insurance coverage speak to another consistent barrier identified by farmers, which was feeling disconnected from healthcare systems and providers by more than physical distance. Farmers identified an incongruity between the structure of the healthcare system, providers’ understanding of the realities of farming, and their own lives. One farmer commented, “Sometimes, like, the barrier can be the appointment. If you’re like, ‘I’m sorry, I can be there 20 min later, but this cow was in a ditch, you know? Or my truck broke down. But I’ll be there in 20 min’. Sometimes they’re like, ‘well, we’ll charge you a cancellation fee”. Many farmers discussed how the unpredictable nature of farming, and farmers’ concern for the health and well-being of their animals, made it hard for farmers to engage with the relative rigidity of overburdened healthcare resources in rural areas.

This disconnect was identified on an interpersonal level as well, with farmers often finding providers to be disinterested in, or unable to comprehend, their lived experience. One dairy farmer said, “Sometimes you tell a doctor something…and they’re like ‘why would you do that?’ and I’m like ‘why wouldn’t you do that?’ You know, like it’s just if they don’t understand it, sometimes they want to tell you (that you) shouldn’t, or you can’t, and so again, farmers don’t want to hear that”. Other farmers shared this experience, commenting that words of support from other farmers were far more impactful than those offered by healthcare providers, whose advice was often incompatible with the realities of farming life. One farmer recounted their experience speaking with a therapist, saying, “You know, she’s like, ‘well, you really just need to take a break…you’ve got to just find time, just pick a day, get away from the farm’. And I’m like, ‘it’s not that easy like that. You’re stressing me out more by telling me to take a day off’”. Another farmer reported finding their interactions with healthcare providers impersonal and felt as though they were ‘just checking a box’ when they asked about mental health or substance use during appointments. Given the close-knit nature of farming communities, impersonal experiences with healthcare providers who also lack farm credibility may undermine farmers’ intentions to utilize resources in the future, exacerbating issues with delayed care within this population.

### 3.5. Stigma

#### 3.5.1. Community Stigma

Stigma was commonly discussed in interviews as a barrier to engaging with resources for mental healthcare, although many interviewees described how stigma impacted male farmers as opposed to their own willingness to discuss mental health with peers or professionals. When asked how community perceptions might impact their willingness to seek out help for a mental health challenge, one interviewee said, “If I were a man in a man’s world, maybe. So being a woman, I would…I think—I think mental health is a big deal, so, I mean, I’ve always felt like that. So they (men) might have a different thought on that”. In fact, three-quarters of respondents indicated that they felt as though women were more open than men to discussion of mental health. Several farmers commented on the prominence of public stigma in rural Southern areas, with one stating, “There’s still a stigma, particularly in rural areas. Even people who want to seek treatment often are afraid to…”. Another commented on using agricultural events as an avenue to reach farmers with messaging about mental health, saying, “…not mental health. Nobody’s going to come up to that table, not with their buddies in the room and then they’re gonna get upset with you if you come to them in public”. Another said, “…we had a segment about mental health, and a lot of the farmers complained because they were like ‘Oh, we don’t need this. You know, this—this is depressing to hear about’”. A third reported challenges getting event planners to entertain a formal discussion of mental health, saying, “But I think they were afraid that nobody would engage in it. Because nobody wants to come out and say you’re having mental issues, or your stress is getting to you”.

#### 3.5.2. Self-Stigma

Self-stigma associated with mental health challenges was less commonly reported. One farmer stated, “You know, I’m like… ‘all the people that have been farming their whole lives, they probably don’t feel this way. I’m being a big baby about this, you know?’” when discussing how animal mortality impacted their own mental health. Another interviewee commented on a perceived self-stigma in others, stating, “I think they feel shame that they need medicine, you know, or don’t want any type of medicine to help them deal with anxiety or stress. I just think there’s a stigma to (it)”. Concerns about stigma in the farming community were connected to social dynamics in small rural communities—one farmer described her experience having a healthcare provider contact her mother after she left an appointment, “…I didn’t like what the dentist had to say. I was a full-grown adult, and they called my mom to tell on me”. Concerns about what others would say, and how quickly word would spread, were acknowledged by many interviewees, although most of the women interviewed clarified that this would not be a barrier for them personally, emphasizing the impact that stigma had on the behavior of others. When discussing the impact of stigma on help-seeking behavior in others in the agricultural community, one farmer said, “I think it’s those perceptions that they’re really entrenched in rural areas and it’s a weakness. I don’t think it is. But that’s the perception—is that it’s a weakness”.

### 3.6. Cultural Norms

Farmers also discussed several cultural norms within the farming community that they connected to the avoidance of medical care in their peers, including prioritization of farm tasks, self-reliance, pride, masculinity, and the minimization of health concerns, particularly those associated with mental health. Multiple farmers identified a pattern of constantly prioritizing agricultural work above personal health and well-being, both in themselves and others, and connected this to a pattern of delaying, or outright avoiding, healthcare. One farmer reported a preference for treating herself with medicinal plants, in lieu of using pharmaceuticals, and attributed this to poor healthcare experiences in the past. In some cases, the prioritization of work demands interacted with other factors, such as healthcare provider disconnection, to further reduce the likelihood of engaging with care. One farmer described this prioritization of work over personal health by saying, “I think they would say, um, I got 100 hours’ worth of work to do this week. Why would I go sit down and give USD 200 an hour to somebody that can’t comprehend what I’m going through?”.

Interviewees connected many of these cultural norms to farmers’ adherence to conventional masculine ideals such as restrictive emotionality, resilience, and self-reliance, which was especially pronounced in older farmers. Several farmers described a ‘tough guy’ persona, with one farmer going so far as to say, “…I think it’s just the stigma that they don’t—they don’t want anybody to know that they are human”. Another farmer described how her father’s restrictive emotionality imprinted on her as a child to the extent that she did not cry publicly at his funeral.

Pride was frequently discussed as a barrier to help-seeking for both mental and physical healthcare needs, with farmers being unwilling to acknowledge that they were struggling with mental health challenges or hearing that they needed to take a break from work to recover. One farmer described how an older farmer in her community, upon losing his driver’s license due to dementia, began to walk wherever he needed to go, traveling on the shoulder of the highway to attend church or buy a cup of coffee.

Several farmers identified a distinct generational gap in willingness to engage with healthcare resources or express emotions, with one saying, “going back to the average age of farmers being older, coming from that generation, mental health was not a topic to be discussed. Like, you just sucked it up, pulled your bootstraps, and rolled on with your life…”. This avoidance of care was also present for physical health concerns, with one farmer describing how, after her father had fallen and severely cut his head on the concrete floor of their barn, he called her to ‘come put a band-aid on his head’ and that “he’s like ‘I’m not getting stitches. I’m not”…So I had to, like, clean it (and) bandage it”. Interview participants commonly acknowledged the impact of these cultural norms on others, even when their own behavior was not directly impacted. Several farmers reported observing this behavior directly in their own families, but others described it more generally, with one commenting, “…I will say, because of the way everybody else is, you learn it from them…it’s easier to just stuff it down and keep going. Because you see everybody else doing that”. Behavior observed in other farmers may reinforce restrictive emotionality and delayed engagement with healthcare in all farmers, even those who are not naturally inclined to do so. This effect may perpetuate generational attitudes towards healthcare, reinforcing a culture of self-reliance and avoidance of care that contributes to poor health outcomes in the farming community.

## 4. Discussion

Female farmers who participated in qualitative interviews identified numerous barriers to engaging with healthcare resources present in their community, including formal healthcare challenges, stigma, and cultural norms, mirroring findings from previous research in the farming community [12,14,17,24]. Many of these barriers overlapped with each other, compounding their impact, and reinforcing farmers’ perceptions of being isolated from healthcare resources and unable to access quality care in a way that met their needs.

Central to many of these barriers was the concept of ‘farm identity,’ where farmers’ connection to their operations ultimately led to the prioritizing of their livestock and crops above their own physical and mental health. Farmers reported considerable time constraints, where the time required to travel to, and engage with, healthcare services was a substantial interruption to their day-to-day routine. In these interviews and prior research in the farming community, farmers reiterate how the demands of farming make it nearly impossible to take hours away from operations for even routine healthcare needs [6,12,24]. Farm identity further impacted interview participants’ perspectives on healthcare providers’ ability to offer culturally sensitive care, with many farmers reporting that they felt as though providers lacked a fundamental understanding of the realities of farm life that invalidated medical advice. Previous research has identified that cultural competence in healthcare providers can bolster help-seeking behavior in farmers, and other researchers such as Hagen et al. and Brumby and Smith have identified the importance of farm credibility in service delivery to the farming community [14,25,26,27]. Continuing education courses, such as Agrability’s FarmResponse, that aim to boost practitioner knowledge of farm life should be expanded among rural practitioners [28]. Beyond increasing farm credibility among healthcare providers, research in the farming community has found that farmers respond positively to messaging delivered by, or tailored for farmers, even on sensitive topics [29,30,31]. The efficacy of using community leaders to deliver mental health messaging within agricultural communities should be further explored.

Research on the female farming population indicates that they often take on additional roles outside of farm operations, which may contribute to the consistent under-prioritization of health, compounding the impact of formal healthcare challenges [32]. Addressing formal healthcare challenges for female farmers should be prioritized, as many female farmers in this sample connected their female identity to greater ease of discussing mental health with their peers, indicating they were less impacted by cultural norms of stoicism and stigma associated with mental health challenges. Mobile clinics are a promising solution to formal healthcare challenges in rural areas, have been used to provide a wide range of services, and may be beneficial for delivering care to female farmers who would not otherwise be able to engage with health services [33]. Localized treatment provision may circumvent the time constraints that are consistently identified as a significant barrier to engaging with healthcare by farmers, and a major factor in the under-prioritization of personal health and well-being.

### Limitations

The findings discussed in this manuscript are not without limitations. While qualitative interviews are useful for capturing the nuances of barriers to care in the farming community, this methodology does limit the number of participants involved in the study due to the logistics of scheduling and traveling to conduct interviews. Furthermore, the data collected during this study were gathered from a convenience sample of producers living in a single Southeastern state, limiting the generalizability of these findings beyond the context of the sample population. Another limitation was the racial homogeneity of this sample, as farmers of racial/ethnic minority status may experience unique barriers to engaging with healthcare compared to their peers.

## 5. Conclusions

While many of the barriers identified by female farmers who participated in these qualitative interviews align with findings of previously conducted research in the farming community, reinforcing the impact of both compounding barriers and ‘farm identity’ on farmers’ help-seeking, the importance of understanding the impact of gender on farmers’ help-seeking should not be under-valued. Gendered differences in experiences of barriers to care, preferences for health-related messaging, and intervention strategies among farmers should be further explored to gain a better understanding of how different avenues may be more effective at connecting farmers to resources depending on their gender. Farm identity may be a useful guide for the development of novel interventions to impact farmer health and well-being, as well as a central element of training to improve cultural competence among rural healthcare providers.

## Figures and Tables

**Table 1:** Participant Demographics. Interviews were conducted in Gordon, Fulton, Franklin, Madison, Newton, Morgan, McDuffie, Meriwether, Harris, Clay, and Mitchell counties. Of these counties, only Fulton, Newton, and Gordon Counties exceed the United States Census Bureau threshold for rurality in terms of population density (<100 individuals per square mile).

Participant	Date	Generation	Race/Ethnicity	Farm Role	Commodity	# of Workers
1	6 September 2023	4th	White	Full-Time Owner/Manager	Dairy Cattle	6
2	18 September 2023	5th	White	Part-Time Owner/Agriculture Education	Beef Cattle, Hay	4 (combination of part and full-time)
3	18 September 2023	4th	White	Full-Time Owner/Manager	Poultry	4 (combination of part and full-time)
4	4 October 2023	5th	White	Full-Time Owner/Manager	Diversified Livestock, Poultry	150
5	4 October 2023	3rd	White	Full-Time Owner/Manager	Citrus, Strawberries, Sunflowers	4–10 (combination of part and full-time)
6	25 October 2023	3rd	White	Full-Time Owner/Manager	Ornamental Crops, Cut Flowers	3–15 (combination of part and full-time)
7	14 November 2023	2nd	Latina	Full-Time Owner/Manager	Certified Organic Vegetable Farm	13 (5 cooperative owners, 8 part-time workers)
8	14 November 2023	2nd	White	Full-Time Owner/Manager	Certified Organic Vegetable Farm	13 (5 cooperative owners, 8 part-time workers)
9	6 December 2023	1st	White	Full-Time Owner/Manager	Fruit, Vegetables, Flowers	2 full time (unspecified seasonal help)
10	6 December 2023	1st	White	Full-Time Owner/Manager	Diverse Produce, Cut Flowers, Herbs	8 (5 full-time, 3 part-time)
11	11 December 2023	1st	White	Full-Time Owner/Manager	Beef Cattle	5 (2 full-time, 3 part-time)
12	13 December 2023	2nd	White	Full-Time Owner/Manager	Beef Cattle, Pork, Row Crops	4–10 (combination of part and full-time)
13	15 December 2023	2nd	African American	Part-Time Owner/Manager	Forestry, Herbs, Fruit and Vegetables, Aquaculture	1 full-time (family assistance and unspecified volunteers)
14	8 January 2024	3rd	White	Full-Time Owner/Manager	Dairy Cattle	5 (1 full-time, 4 part-time)
15	6 February 2024	2nd	White	Part-Time Owner/Manager	Beef Cattle	2 full-time
16	28 February 2024	3rd	African American	Full-Time Owner/Manager	Vegetables, Fruit, Herbs	1 full time (unspecified volunteers and day labor)

**Table 2:** Quotes About Barriers to Care.

Theme	Concepts	Quotes
Formal Healthcare Challenges	Time Constraints	“I mean, I think the time, like the time demands of farming make it hard to weave self-care in, especially in the height of the season. Like it’s just- it’s hard to take a half day off or a whole day off if, you know, the team’s got a really hard day and you’ve got a lot to do”. [Participant 8, Certified Organic Vegetable Farmer]
	Lack of Insurance and Cost of Accessing Care	“And there’s just so much red tape with everything, you know, that even just keeping up with the paperwork, there’s a program that provides free health insurance to farmers through [Organization Name]. But like I checked one box wrong. So now there’s a denial letter and I have to reapply. And it’s just like you give up because it’s so exhausting”. [Participant 16, Fruit and Vegetable Farmer]
	Provider Disconnection and Cultural Competence	“…when you can help that animal. To be like ‘oh, sorry, I’m going to the dentist’. That’s like leaving if your kid busted their head open on the sidewalk. You’re going to stop and take your kid where it needs to go… And if farmers are already experiencing financial distress, and then you tell them ‘well we’re going to charge you a cancellation fee’. Well, I’m never going to make the appointment”. [Participant 14, Dairy Cattle Farmer]
		“It’s just extremely complicated to talk to someone who doesn’t understand. Like, I’m better off talking to my friend that farms down the road because, you know, if I lose a calf, then I feel really bad because, you know, you feel really guilty, really guilty. And he’s like, ‘well, you did—You did all you could’. Like that makes me feel better for him to say that. But if a therapist that doesn’t understand, is like ‘you did all you could’ you know, it just doesn’t—It doesn’t—It’s not the same”. [Participant 15, Beef Cattle Farmer]
		“I just had my physical yesterday and they ask you ‘‘have you been depressed or have you lost interest in doing things that you used to love to do?’ They ask you that when you first go in—and I sat there and I was like, you know, they don’t even look at you when they’re asking that… They don’t—it’s just a form they’re checking off”. [Participant 5, Citrus Farmer]
Stigma	Community Stigma	“There’s still a stigma, particularly in rural areas. Even people who want to seek treatment often are afraid to, even within our churches we are often told to suck it up, lean on God, suck it up. It doesn’t matter if you can really handle it or not… people will say, God doesn’t give you more than you can handle. Oh, yes, he does. He absolutely does”. [Participant 6, Ornamental Crop Farmer]
		There was a thing we were trying to put on through the chamber for the Cattlemen’s Association that we were having to choose the topic. Do we want to do the topic on mental health or do we want to do like lifesaving things you can do on the farm, like having tourniquets and stuff? Immediately there was no talking about the mental health. Nobody wanted to go there. [Participant 11, Beef Cattle Farmer]
	Self-Stigma	“I think there’s um, I think they feel shame that they need medicine, you know, or don’t want any type of medical, any type of medicine to help them deal with anxiety or stress”. [Participant 2, Beef Cattle and Hay Farmer]
Cultural Norms	Prioritization of Work	“I think sometimes we don’t take care of ourselves and go to the doctor or the dentist or whatever like we should, because we are spending all of our time working. And, you know, we really don’t leave the farm much because we’re—we’re working so much. I think that’s an issue for us and probably for other farmers, too”. [Participant 9, Fruit and Vegetable Farmer]
	Restrictive Emotionality	“He wouldn’t have sought help for that (mental health). ‘Ain’t no way, I can get through this’. You know, and he kind of reflected that on me. No, you can’t cry and you can’t do this. You can’t do that, you know? So at his funeral, I didn’t shed a tear. You know, I went in another room (and) fell apart”. [Participant 5, Citrus Farmer]
	Pride	“Farmers are very proud types, so being vulnerable enough to go. Um that would probably be one. They don’t want to exhibit that something’s wrong with them. And they don’t want a doctor to tell them that they can’t do (something), because if the doctor tells them they can’t do (something), they’re going to find a way anyway”. [Participant 14, Dairy Cattle Farmer]
		“Down [highway number] to church, to the gas station to get his coffee in the morning… But you tell a farmer they can’t—they’ll find a way. So they don’t want to hear that from a doctor, so they typically won’t go”. [Participant 14, Dairy Cattle Farmer]

## Data Availability

The anonymized data supporting the conclusions of this article will be made available by the authors on request.

## Supplementary Materials

The following supporting information can be downloaded at: https://www.mdpi.com/article/10.3390/ijerph21091130/s1, File S1: Interview Script.
