# Supplementary material for: “They Can’t Possibly Understand What I’m Going Through”: Female Farmers’ Perspectives on Barriers to Care in Georgia
Source: Int J Environ Res Public Health. 2024 Aug 27;21(9):1130. doi: 10.3390/ijerph21091130 (PMC11431051; doi:10.3390/ijerph21091130)
Supplement: Supplementary file 1 [file ijerph-21-01130-s001.zip › ijerph-3079950-supplementary.pdf]

Interview Script

FARMS: Finding Appropriate measurements for Rural Mental health and Substance use

The University of Georgia Cooperative Extension, Archway Partnerships, and College of Public health want to promote well-being and help farming communities support each other during times of stress. We are asking you to take part in an interview conducted by Dr. Christina Proctor, College of Public Health, cproctor@uga.edu, 706-583-8184. The purpose of this study is to find out what is influencing farmer's health and identifying barriers to improving health in rural Georgia farmers. We also want to better understand farm stress in Georgia and identify effective ways to provide information on health and stress to farming communities.

Because you are a full-time farmer, we think you have valuable knowledge about the state of farming and factors influencing farmer stress. We are asking you to complete this 30-35 minute interview. The interview is totally voluntary and you can stop at any point. If any of the questions make you uncomfortable, you can skip them if you like. Your responses will help us understand the factors influencing farmer's health in Georgia and best ways to communicate information to farming communities on improving well-being and managing stress. This interview is confidential and responses will only be reported as a summary of the data. If you have any complaints or questions about your rights as a research volunteer, contact IRB at 706-542-3199 or by email at IRB@uga.edu.

Screening questions:

Do you agree to participate in this interview and will you allow us to record this conversation?

Does at least 75% of your income come from farming?

Interview questions:

1. *What type of farming do you do?*
    a. *How many people work on your farm?*
2. *How long have you been a farmer?*
3. *Can you tell us about your family's history with farming?*
4. *What are major stressors associated with farming?*
    a. Do you think female farmers experience unique stressors? If so, what are they?

5. *Do you feel that you have been treated differently as a female in a male dominated industry? If so, how?*
6. *Do you feel like you have had to act differently, or do things you wouldn't normally do, being a female in a male-dominated industry? If yes, please provide examples.*
7. *What ways have you seen farmers/farmworkers coping with those stressors?*
    a. *If substance use is disclosed, probe about type but not volume*
8. *Have you seen any negative health effects in farmers/farmworkers because of their coping strategies?*
    a. *Inquire Physical Health*
    b. *Inquire Mental Health*
9. *Have you experienced any negative health effects as a result of farming or farmwork?*
    a. *Inquire Physical Health*
    b. *Inquire Mental Health*
10. *Have your workers experienced any negative health effects as a result of farming or farming?*
11. *If you have experienced any negative health effects as a consequence of farming, were you able to receive treatment?*
    a. *What barriers did you find in accessing treatment?*
    b. *What avenues for treatment did you find helpful?*
    c. *Would you feel comfortable discussing mental health issues that you are experiencing with your family doctor or current healthcare provider?*
        i. *Why or why not*
12. *How does the way that other members of your community view mental health issues impact your likelihood of seeking treatment for issues with mental health you may be experiencing?*
13. *What stressors associated with farming do you think have an impact on farmers' mental health?*
    a. *What coping strategies have you observed other farmers and farm workers using to deal with mental health issues?*
14. *Do you think farmers or farm workers are open to discussion of stressors and their impact on mental health?*
    a. *If not, what barriers exist to having those discussions?*

15. *Have you heard of any farmers using substances to alter their mood to deal with stressors associated with farming?*
    a. *Which substances?*
16. *Do you think farmers would be receptive to receiving information about stress, substance use, and broadly, mental health?*
    a. *If "no" then why*
    b. *What avenues do you think would be the most effective at providing that information to farmers and farmworkers?*
17. *Is there an existing treatment option for mental health issues or substance abuse issues in your area that you are aware of?*
    a. *If "no" then what intervention do you think would have the most impact?*
    b. *Would you feel confident in using that treatment option, if you or a family member was experiencing issues with substance abuse or mental health?*
18. *What are your concerns for the future of this farm and farming generally?*
